# An eHealth Prevention Program for Substance Use, Sexual Assault, and Sexual Risk Behaviors for Adolescents in Primary Care: Pilot Feasibility Randomized Controlled Trial of Teen Well Check

**DOI:** 10.2196/50833

**Published:** 2023-11-02

**Authors:** Amanda K Gilmore, Kennicia Fortson, K Nicole Mullican, Grisel García-Ramírez, Anna Hutchins, Alyssa M Bartlett, Holly C Gooding, Elizabeth Wallis, Sharon Levy, Kenneth J Ruggiero, Debra Kaysen, Carla Kmett Danielson, Robert Platner, April Hartman, Shannon Self-Brown

**Affiliations:** 1 Department of Health Policy & Behavioral Sciences School of Public Health Georgia State University Atlanta, GA United States; 2 National Center for Sexual Violence Prevention, Mark Chaffin Center for Healthy Development School of Public Health Georgia State University Atlanta, GA United States; 3 School of Social Work University of Georgia Athens, GA United States; 4 School of Medicine Duke University Durham, NC United States; 5 Department of Pediatrics Emory University School of Medicine Atlanta, GA United States; 6 Department of Pediatrics Medical University of South Carolina Charleston, SC United States; 7 Division of Addiction Medicine Boston Children’s Hospital Boston, MA United States; 8 College of Nursing Medical University of South Carolina Charleston, SC United States; 9 Department of Psychiatry & Behavioral Sciences Medical University of South Carolina Charleston, SC United States; 10 Division of Public Mental Health & Population Sciences, Department of Psychiatry & Behavioral Sciences Stanford University Medical Center Stanford, CA United States; 11 Old Fourth Ward Pediatrics Atlanta, GA United States; 12 Department of Pediatrics Medical College of Georgia Augusta University Augusta, GA United States

**Keywords:** prevention, adolescents, eHealth, drug use, sexual assault, sexual health

## Abstract

**Background:**

Substance use, sexual assault, and sexual risk behaviors are common among adolescents and are interrelated. Nearly 1 in 5 adolescents use substances before sexual encounters, placing these young people at risk for both sexual assault and sexual risk behaviors. Primary care visits present a unique opportunity to address multiple health risk behaviors.

**Objective:**

*Teen Well Check* is a web-based integrated prevention program for substance use, sexual assault, and sexual risk behaviors with demonstrated usability and acceptability among patients and providers. The aim of this study was to conduct a pilot randomized controlled trial to assess feasibility.

**Methods:**

Adolescents (n=123) aged 14 to 18 years from diverse backgrounds were recruited from primarily Medicaid-serving pediatric primary care clinics. Participants completed a baseline survey; were randomized to receive *Teen Well Check* or an assessment-only control; and completed 1-, 3-, and 6-month follow-up surveys. Feasibility was assessed in terms of recruitment and retention rates. Preliminary changes from baseline to follow-up periods were examined separately in the *Teen Well Check* and control conditions.

**Results:**

We recruited 123 participants (*Teen Well Check*: n=61, 49.6%; control: n=62, 50.4%). Of the 61 participants assigned to the *Teen Well Check* condition, 55 (90%) completed the full program and viewed all intervention content. Of the 123 participants, 105 (85.4%) were retained across at least 1 follow-up period, and there was no difference in follow-up rates between the conditions (*χ^2^_1_*=0.6; *P*=.43). The completion of *Teen Well Check* took an average of 6.2 (SD 5.8) minutes. Preliminary analyses revealed that there were significant reductions in perceived peer norms (descriptive norms) for substance use before sex across follow-ups among participants in the *Teen Well Check* condition (*P*=.001 from baseline to 6 months), whereas there were significant increases among participants in the control condition (*P*=.003 from baseline to 6 months). In addition, there were nonsignificant reductions in substance misuse risk from baseline to the 6-month follow-up among participants in the *Teen Well Check* condition (*P*=.16).

**Conclusions:**

These findings support the feasibility of *Teen Well Check* delivery within pediatric primary care clinics. A randomized clinical trial is needed to assess efficacy.

**Trial Registration:**

ClinicalTrials.gov NCT3489434; https://www.clinicaltrials.gov/study/NCT03489434

## Introduction

### Background

Substance use is common among adolescents, and trends show increased use during the COVID-19 pandemic [1]. According to the 2022 Monitoring the Future data that describe substance use among 8th, 10th, and 12th graders [1], 15.2% to 51.9% of adolescents used alcohol in the past year, 8.3% to 30.7% used cannabis, 12.0% to 27.3% vaped, 6.1% to 16.8% used cigarettes, and 4.9% to 8% used illicit drugs other than cannabis. Sexual assault is also common among adolescents. The National Intimate Partner and Sexual Violence Survey found that 26.8% of adult women and 3.8% of adult men reported experiencing completed or attempted rape in their lifetime [2]. Of those assaulted, 34.9% of the women and 29.8% of the men experienced their first attempted or completed rape between the ages of 11 and 17 years [2]. Adolescents also engage in sexual risk behaviors; of the 30% who indicated that they have had sex, 48% reported not using a condom the last time they had sex [3].

Substance use, sexual assault, and sexual risk behaviors are interrelated. Nearly 1 in 5 adolescents reports using substances before sexual encounters [4], placing these young people at risk for both sexual assault and sexual risk behaviors owing to impairments in decision-making [5]. Inversely, the experiences of sexual assault increase the risk for engaging in substance use as a coping mechanism [6]. Given the associations among substance use, sexual assault, and sexual risk behaviors, integrated prevention programming that addresses all 3 content areas is imperative for adolescents.

Technology-based personalized normative feedback interventions are commonly used to prevent and reduce drug use and related behaviors among youth and young adults and result in small effect sizes [7]. Personalized normative feedback interventions are based on social norms theory [8] and aim to correct 2 common misconceptions about risk and protective behaviors. Individuals tend to overestimate the rate at which similar-aged and same-gender peers engage in risk behaviors, whereas they underestimate the rate of protective and health behaviors; for example, youth and young adults overestimate substance use [9] and underestimate bystander intervention behavior [10] and condom use [11]. According to social norms theory [8], individuals engage in behavior to match social norms. In fact, descriptive norms, or perceptions of peer norms, are associated with substance use, sexual assault, and sexual risk behaviors among youth and young adults [10,12,13]. Therefore, correcting these common misperceptions should decrease risk behavior (eg, substance use before sex).

Primary care is one of the very few settings in which adolescents receive routine preventive health care. Implementing new prevention programming during routine primary care appointments may be feasible because adolescents and their guardians are seeking out and expecting prevention messages in this setting. Primary care providers are expected to screen for health risk at every well visit. Furthermore, the delivery of programs in primary care settings allows adolescents and their guardians natural contact with providers they trust and with whom they have a relationship and provides an opportunity to ask follow-up questions about the health risk behaviors targeted in the prevention programs. Bright Futures guidelines [14] outline 12 health promotion categories and the developmental age at which these should be addressed. As health promotion categories increase, primary care providers often feel pressure to balance screening and counseling on these behaviors owing to time constraints. Each of the 12 health promotion categories has several topics that need to be covered for adolescents. To give an example, within a single visit, providers are expected to counsel adolescents on safety related to driving, violence (which includes sexual violence and other forms of violence), suicide, gangs, and sports—and safety is only 1 of the 12 health promotion categories. Providing an evidence-based technology-based screen and brief intervention for even one of these health promotion categories could reduce provider burden while providing adolescents with needed evidence-based prevention.

Technology-based interventions may be particularly useful in this setting because they can deliver efficient standardized screening and brief interventions on a wide range of behaviors in a modality that is preferred by adolescents [15,16]. Furthermore, technology-based interventions allow for potential integration into primary care without added burden to primary care providers. Specifically, technology-based interventions can be implemented within typical clinic flow while adolescents are either in the waiting room or waiting for their physician in an examination room. They can also be integrated into electronic medical records to reduce documentation burden for primary care providers.

### This Study

Teen Well Check (TWC) is an eHealth program grounded in social norms theory that uses a motivational interviewing approach and provides (1) personalized feedback on norms for substance use as well as substance use before sex tailored by age and gender [17]; (2) sample language to foster communication related to substance use, sexual assault resistance and bystander intervention, and sexual communication; and (3) psychoeducation on teen substance use, sexual assault, and sexual risk behaviors, including the effects of substances on the brain, alternative coping activities to engage in instead of substance use, definitions of sexual consent and sexual assault, how to be an active bystander in a potential sexual assault situation, how to effectively engage in asking for and giving consent and other sexual communication skills, information about sexually transmitted infections, and how to effectively use condoms—all delivered in an interactive format using comic art. TWC has been assessed as both usable and acceptable among adolescents and providers [17]. However, it is unclear whether it is feasible for this program to be integrated into preventive health care in primary care settings. Therefore, the aim of this study was to assess the feasibility of the recruitment and retention of participants in a randomized controlled trial. We established numerous benchmarks for feasibility. First, we considered an enrollment rate of >50% to be supportive of feasibility. Second, for those assigned to TWC, we considered >75% of the participants viewing all program content to be supportive of feasibility. Third, we considered pooled retention rates of >80% across all follow-up periods to be supportive of feasibility. We also examined preliminary changes over 1-, 3-, and 6-month follow-up periods in substance misuse risk and descriptive norms for substance use before sex.

## Methods

This paper reports the results of a randomized controlled trial designed to assess the feasibility and the effect size of TWC among adolescent primary care patients aged 14 to 18 years.

### Participants

We recruited adolescents presenting for a routine medical visit to 1 of 2 primary care clinics in the Southeastern United States between September 28, 2021, and April 7, 2022, and the follow-up surveys ended on October 3, 2022. Although social distancing mandates from the COVID-19 pandemic had been lifted, there were still restrictions in medical settings during this time in terms of mask requirements in pediatric clinics and the elimination of the use of waiting rooms (waiting occurred outside of the clinics, eg, in the car). Participants were eligible to participate in the study if they (1) were aged 14 to 18 years, (2) were able to read English, and (3) did not have an intellectual or cognitive disability.

### Recruitment, Randomization, and Assessments

Eligible participants (and the guardians of minors) were invited to meet with a research assistant in person at the clinic, over the telephone, or in a videoconference meeting to discuss the research, complete an eligibility screen, and provide consent. Interested adolescents completed a brief eligibility screen (2-3 min) on a tablet computer or on the web. Staff informed eligible participants and guardians about the sensitive nature of the prevention program and explained that reporting and potentially disclosure to their provider and the principal investigator would occur if there was a sexual assault history that had not previously been reported or if there was imminent risk of suicide. After reviewing this material with participants, staff obtained informed consent from those aged >18 years or adolescent assent and parental consent in the case of those aged <18 years.

All consented participants were sent a link to the baseline survey and were randomized to either intervention (TWC) or control (assessment only) upon survey completion on a 1:1 allocation basis. Randomization was conducted in Qualtrics using block randomization. The principal investigator was blinded to condition assignment for the duration of the study.

Participants in the intervention arm were asked to complete a brief (5 min) postintervention survey. All participants were sent 30-minute follow-up assessment batteries 1, 3, and 6 months after their baseline survey.

All procedures outlined in this paper are in accordance with the CONSORT (Consolidated Standards of Reporting Trials; 2010) and the CONSORT extension to pilot trials (2016) recommendations [18]. This trial is registered at ClinicalTrials.gov (NCT3489434).

### Ethical Considerations

All study procedures were approved by the university institutional review board (Georgia State University, institutional review board study ID H21524). Research staff reviewed the informed consent form and study procedures with participants and staff obtained informed consent from those aged >18 years or adolescent assent and parental consent in the case of those aged <18 years. Participants could opt out at any point of the study. Hard copies of data were stored in a locked office and digital data were stored on encrypted university servers that required dual-identity log-in and was only available to members of the study team. Participants’ data were stored using a study identification code. Study identification codes were linked in a document on a password-protected file stored on encrypted university servers that required dual-identity log-in and was only available to members of the study team. Participants received a gift card for completing the eligibility screen and each of the assessment batteries for a possible total compensation of US $195.

### Intervention

#### TWC Modules

TWC is a web-based program that consists of 3 modules to address substance use, sexual risk behavior, and sexual assault with a motivational interviewing approach. This program was presented to participants immediately after the baseline survey. The modules were presented in a sequential manner, and the content was grounded in evidence-based literature, social norms theory, and motivational interviewing. TWC addresses the following health promotion categories within the Bright Futures guidelines [14]: (1) promoting mental health, (2) promoting healthy sexual development and sexuality, and (3) promoting safety and injury prevention. As recommended in the Bright Futures guidelines [14], TWC includes the CRAFFT (car, relax, alone, forget, friends, trouble) tool [19] to assess for substance misuse risk, provides psychoeducation on adolescent sexual decision-making, and provides content to reduce the risk of sexual violence.

#### TWC Modules: Substance Use

Participants were provided information about 1 substance type in this module: vaping, cigarettes, or JUUL; alcohol; cannabis or marijuana; prescription opioids; or other illegal drugs. If a participant reported use of 1 substance (eg, alcohol) in the baseline survey, they learned about that substance. Participants who reported no substance use or use of >1 substance in the baseline survey chose which substance they wanted to learn about. The content included (1) personalized normative feedback based on gender identity and age in relation to 1 substance, (2) psychoeducation on the impact of substance use on brain development, and (3) negative consequences of substances reported in the baseline survey. The module also included a concise video from the National Institute on Drug Abuse [20] that covered the effects of substance use on brain anatomy and physiology.

#### TWC Modules: Sexual Risk

The content included personalized normative feedback based on gender identity and age in relation to the percentage of adolescents who have been tested for sexually transmitted infections and the percentage of adolescents who chose to not use substances before engaging in sexual behavior. The psychoeducation in this module was centered around sexual communication, condom use, and the impact that substances can have on sexual communication.

#### TWC Modules: Sexual Assault

This module included comic art–style scenarios of potential sexual assault situations to equip participants with the knowledge and skills necessary to respond appropriately as a potential person at risk of experiencing sexual assault, perpetrator, or bystander. There were 3 different scenarios including a scenario about nonconsensual sexting, sexual contact in a school hallway, and sexual pressure at a house party. Participants selected 1 of the 3 scenarios to learn about how they could respond in that situation. Moreover, this module featured psychoeducation regarding sexual consent and sexual communication, including boundary setting and asking others about their comfort and boundaries. It also reviewed the effects of substance use on an individual’s capacity to give or receive consent during sexual encounters.

Participants randomized to the control condition received assessment batteries only.

### Measures

#### Feasibility

First, feasibility was assessed by the number of eligible participants who enrolled in the study. Second, feasibility was assessed by examining intervention completion. *Intervention completion* was categorized in 3 ways: participants who completed all 3 modules were labeled as *completers*, those who completed 1 or 2 modules were labeled as *partial completers*, and those who did not complete any of the modules were labeled as *noncompleters*. Furthermore, time to complete the intervention was assessed in minutes. Third, we considered pooled retention rates of >80% across all follow-up periods to be supportive of feasibility. We also examined preliminary changes over 1-, 3-, and 6-month follow-up periods in substance misuse risk and descriptive norms for substance use before sex.

#### Substance Use and Risk of Substance Misuse

These were assessed at baseline using the National Institutes of Health Screening to Brief Intervention tool [21]. Participants were asked to indicate their experiences of substance use in the past year on a scale of 1 to 4 (1=never, 2=once or twice, 3=monthly, and 4=weekly or more) for tobacco, alcohol, marijuana, prescription drugs they were not prescribed (eg, pain medication or Adderall), illegal drugs (eg, cocaine or ecstasy), inhalants (eg, nitrous oxide), herbs or synthetic drugs (eg, salvia, *K2*, or bath salts), and vaping or JUUL. Participants’ responses were given a dichotomous score (0=no use or 1=any use) to characterize the sample. The reliability of the Screening to Brief Intervention ranged from Cronbach α=.71 to α=.73 across the assessment periods. The CRAFFT [19] was used to assess the risk of substance misuse at baseline and at 1-, 3-, and 6-month follow-ups. This tool assesses substance use using 6 items designed to identify substance use, substance-related riding or driving risk, and substance use disorder (SUD) among youth aged 12 to 21 years. A score of 0 to 1 indicates low risk of SUD, and a score of ≥2 indicates the potential of a significant alcohol or other substance misuse problem. The test-retest reliability of the CRAFFT across 6 months is not known. However, the tool is widely used in pediatric clinics owing to its predictive validity of SUDs at 1-, 2-, and 3-year follow-ups [22]. The Cronbach α values for the CRAFFT in this study ranged from .73 to .97 across the assessment periods, demonstrating acceptable to good reliability.

#### Descriptive Norms for Substance Use Before Sex

Participants rated the proportion of teens that they believe use substances before sex between 0% and 100%. This was assessed at baseline; after the intervention (only for those assigned to the TWC condition); and at 1-, 3-, and 6-month follow-ups.

### Data Analysis

All analyses were conducted in SPSS software (version 28.0; IBM Corp). The primary outcomes of the study were related to feasibility, which was assessed using recruitment and retention rates. Similar to other rigorous pilot feasibility randomized controlled trial designs [23], the feasibility of recruitment was established pragmatically, and target enrollment was determined to be 120 participants over a 9-month recruitment period. We considered an enrollment rate of eligible participants of >50% supportive of feasibility. We considered >75% of the participants viewing all program content supportive of feasibility. A meta-analysis of randomized controlled trials focused on substance use found the median retention rate to be 80% [24]. Therefore, a retention rate of ≥80% was considered supportive of feasibility.

The secondary outcomes examined in this study include an examination of changes from baseline to follow-up time points within the conditions. As small effect sizes are expected for technology-based personalized normative feedback interventions [7] and the current sample size was not powered to detect small effect sizes in outcomes, *P* values to test both significant differences and effect sizes were examined. For the purposes of the pilot, a statistically significant difference at the significance level of α=.05 or an effect size of at least 0.2 (small) was considered a preliminary finding. Wilcoxon signed rank tests were conducted to assess preliminary changes in substance misuse risk between baseline and follow-up periods by condition, and the effect size for the Wilcoxon signed rank tests was evaluated using *r*. Paired samples *t* tests (2-tailed) were conducted to assess changes in descriptive norms for substance use before sex over time, and Cohen *d* was used to assess effect size. We did not conduct α corrections owing to the underpowered nature of pilot studies.

## Results

### Overview

Most of the participants identified as Black or African American (117/123, 95.1%), and 18.7% (23/123) identified as belonging to a sexual minoritized group. Participants were evenly randomized to the TWC (61/123, 49.6%) or control condition (62/123, 50.4%). Of the 123 participants, 39 (31.7%) reported using a substance in the past year, 27 (22%) reported ever engaging in sex, and 5 (4.1%) reported using substances before sex in the past 90 days. Full sample demographics are presented in Table 1.

**Table 1:** Demographic and baseline variables by condition.

Demographic and baseline variables			Intervention (n=61)		Control (n=62)		Total (n=123)
Age (years), mean (SD)			15.62 (1.39)		15.63 (1.24)		15.63 (1.31)
**Gender, n (%)**							
	Boy	31 (50.8)		27 (43.5)		58 (47.2)	
	Girl	28 (45.9)		34 (54.8)		62 (50.4)	
	Nonbinary	2 (3.3)		1 (1.6)		3 (2.4)	
**Race, n (%)**							
	Asian	0 (0)		1 (1.6)		1 (0.8)	
	Black or African American	59 (96.7)		58 (93.5)		117 (95.1)	
	White	2 (3.3)		1 (1.6)		3 (2.4)	
	Not listed or prefer not to answer	0 (0)		2 (3.2)		2 (1.6)	
**Ethnicity, n (%)**							
	Hispanic or Latine	1 (1.6)		4 (6.5)		5 (4.1)	
	Not Hispanic or Latine	46 (75.4)		50 (80.6)		96 (78)	
	Prefer not to answer	14 (23)		8 (12.9)		22 (17.9)	
**Sexual orientation, n (%)**							
	Heterosexual	46 (75.4)		40 (64.5)		86 (69.9)	
	Gay	1 (1.6)		2 (3.2)		3 (2.4)	
	Lesbian	1 (1.6)		3 (4.8)		4 (3.3)	
	Bisexual	4 (6.6)		10 (16.1)		14 (11.4)	
	Questioning	0 (0)		2 (3.2)		2 (1.6)	
	Not listed or prefer not to answer	9 (14.7)		5 (8)		14 (11.4)	
**S2BI^a^: substance use risk by type^b^, n (%)**			19 (31.1)		20 (32.3)		39 (31.7)
	Alcohol	8 (13.1)		8 (12.9)		16 (13)	
	Cannabis	8 (13.1)		12 (19.4)		20 (16.3)	
	Tobacco or vaping	5 (8.2)		5 (8.1)		10 (8.1)	
	Prescription drug misuse	8 (13.1)		8 (12.9)		16 (13)	
	Illegal drugs	2 (3.2)		1 (1.6)		3 (2.4)	
Ever engaged in sex^c^, n (%)			14 (24.6)		13 (21.7)		27 (23.1)
CRAFFT^d^ risk^c^, n (%)			6 (9.8)		7 (11.3)		13 (10.6)
Substance use before sex in past 90 days^b^, n (%)			1 (1.7)		4 (6.5)		5 (4.1)

^a^S2BI: Screening to Brief Intervention.

^b^Includes 3 risk categories of no reported use, lower risk, and higher risk. Given the small percentages of use among this population, lower risk and higher risk were collapsed into any risk.

^c^Percentages were derived from valid responses, given missing data.

^d^CRAFFT: car, relax, alone, forget, friends, trouble.

### Feasibility: Recruitment and Retention

We enrolled 123 participants in the study in 6 months. Of the 193 adolescents who were screened for eligibility, 4 (2.1%) were ineligible. Of the 189 eligible participants, 3 (1.6%) declined to participate, 2 (1.1%) withdrew, and 5 (2.6%) were withdrawn by the researchers owing to a technical error. A total of 179 adolescents were eligible and willing to participate; however, 56 (31.3%) did not complete the baseline survey and were not randomized to a study condition. Therefore, among those eligible, 68.7% (123/179) enrolled in the study and were randomized to a treatment condition. Of the 61 participants randomized to the TWC condition, 55 (90%) were completers, 1 (2%) was a partial completer, and 5 (8.2%) were noncompleters. The full intervention took an average of 6.2 (SD 5.8) minutes to complete. Completion times ranged from 2 to 28 minutes.

**Figure 1:**
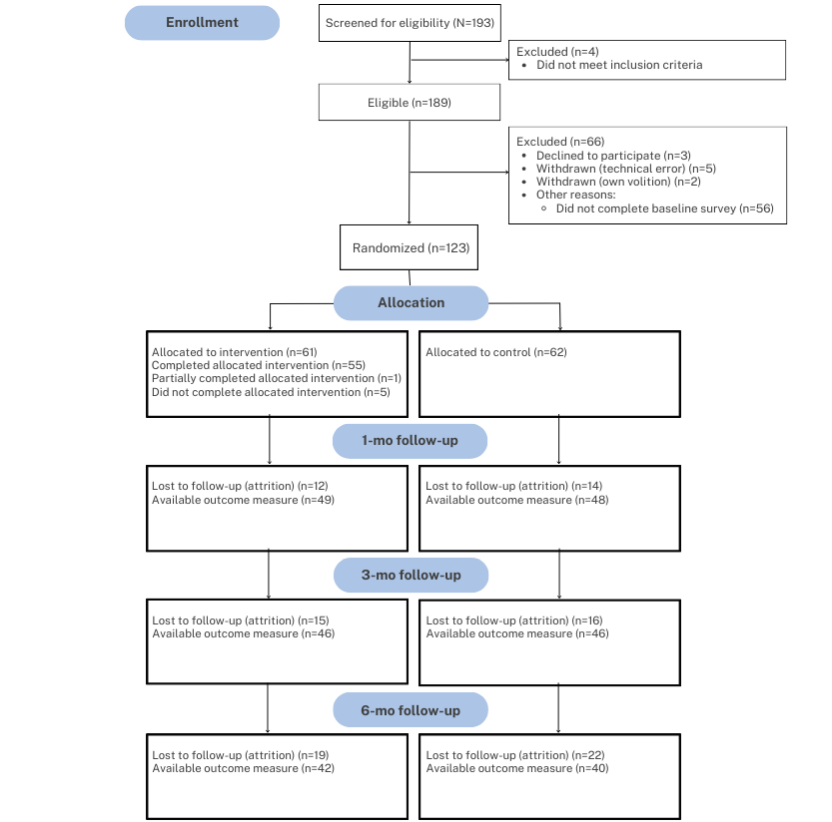
CONSORT (Consolidated Standards of Reporting Trials) diagram. The number of participants analyzed is included in Table 3.

In terms of retention, 85.4% (105/123) completed at least 1 follow-up survey, with 78.9% (97/123), 74.8% (92/123), and 66.7% (82/123) completing the 1-, 3-, and 6-month follow-up surveys, respectively (Figure 1). There was not a significant difference between the conditions in follow-up rates (*χ^2^_1_*=0.6; *P*=.43; Table 2).

**Table 2:** Descriptive statistics for follow-up rates, substance misuse risk, and descriptive norms for substance use before sex.

		Intervention (n=61)	Control (n=62)	Difference by condition				
				Chi-square (*df*)	Cramer *V*	*t* test (*df*)	Cohen *d*	*P* value
Pooled follow-up rates, n (%)		50 (82)	53 (86)	0.3	0.048	N/A^a^	N/A	N/A
**Substance misuse risk (CRAFFT^b^ score of ≥2), n (%)**								
	Baseline	6 (10)	7 (11)	0.1 (1)	0.024	N/A	N/A	.79
	1-mo follow-up	3 (5)	4 (7)	0.2 (1)	0.041	N/A	N/A	.69
	3-mo follow-up	3 (5)	6 (10)	0.9 (1)	0.104	N/A	N/A	.35
	6-mo follow-up	1 (2)	4 (7)	1.8 (1)	0.159	N/A	N/A	.17
**Descriptive norms for substance use before sex, mean (SD)**								
	Baseline	*44.69 (19.07)* ^c^	*21.55 (26.40)*	N/A	N/A	*5.374 (115)*	*0.995*	<.001
	After the intervention	36.38 (26.40)	N/A	N/A	N/A	N/A	N/A	N/A
	1-mo follow-up	30.18 (26.31)	21.70 (23.22)	N/A	N/A	1.594 (85)	0.342	.12
	3-mo follow-up	29.72 (27.77)	24.26 (26.48)	N/A	N/A	0.927 (83)	0.201	.36
	6-mo follow-up	30.29 (23.70)	34.44 (27.01)	N/A	N/A	−0.730 (78)	0.163	.47

^a^N/A: not applicable.

^b^CRAFFT: car, relax, alone, forget, friends, trouble.

^c^Italicization indicates values that met the significance threshold (*P*<.05).

### Intervention Engagement

Most of the participants learned about cannabis (21/61, 34%), followed by illegal drugs (17/61, 28%), tobacco or vaping (13/61, 21%), alcohol (3/61, 5%), and prescription drug misuse (2/61, 3%). Sexual risk behaviors selected were split, with 38% (23/61) choosing the sexting scenario, 30% (18/61) choosing the scenario about a house party, and 23% (14/61) choosing the scenario about sexual contact in the school hallway.

### Preliminary Changes in Outcomes Among Participants by Condition

There were no statistically significant changes in SUD risk (CRAFFT) across time in either the intervention condition or the control condition. However, there was a nonsignificant decrease from baseline to 6-month follow-up in the number of participants with a positive CRAFFT score in the intervention arm (from 6/61, 10% at baseline to 1/61, 2% at 6-month follow-up; *Z*=−1.414; *r*=−0.236; Table 3). Substance misuse risk in the control condition did not have changes from baseline to follow-up that reached a small effect size or significance (Table 2).

There was a statistically significant difference between the TWC (mean 44.69, SD 19.07) and control (mean 21.55, SD 26.40) conditions at baseline for descriptive norms for substance use before sex (*t*_115_=5.374; *P*<.001; Cohen *d*=0.995). There was a statistically significant decrease in descriptive norms for substance use before sex among those in the intervention condition between baseline (mean 44.69, SD 19.07) and after the intervention (mean 36.38, SD 26.40; *t*_51_=1.977; *P*=.053; Cohen *d*=0.274), baseline (mean 44.69, SD 19.07) and 1-month follow-up (mean 30.18, SD 26.31; *t*_42_=3.550; *P*<.001; Cohen *d*=0.541), baseline (mean 44.69, SD 19.07) and 3-month follow-up (mean 29.72, SD 27.77; *t*_40_=2.624; *P*=.01; Cohen *d*=0.410), and baseline (mean 44.69, SD 19.07) and 6-month follow-up (mean 30.29, SD 23.70; *t*_38_=2.951; *P*=.005; Cohen *d*=0.516; Table 3). Furthermore, there was a statistically significant increase in descriptive norms for substance use before sex between baseline (mean 21.55, SD 26.40) and 6-month follow-up (mean 34.44, SD 27.01) among those in the control condition (*t*_38_=−2.951; *P*=.003; Cohen *d*=−0.473).

**Table 3:** Changes in substance misuse risk and descriptive norms for substance use before sex over time by condition.

		Intervention (n=61)							Control (n=62)							
		Values, n^a^ (%)	*Z* score	Effect size		*t* test (*df*)	*P* value	Values, n (%)		*Z* score	Effect size			*t* test (*df*)		*P* value
				*r*	Cohen *d*						*r*	Cohen *d*				
**Substance misuse risk (CRAFFT^b^ score of ≥2)**																
	Baseline to 1-mo follow-up	45 (74)	−0.577	−0.086	N/A^c^	N/A	.56	38 (61)		−0.378	−0.056	N/A	N/A		.71	
	Baseline to 3-mo follow-up	40 (66)	−0.577	−0.091	N/A	N/A	.56	43 (69)		0.000	0.000	N/A	N/A		<.99	
	Baseline to 6-mo follow-up	36 (59)	−1.414	−0.236	N/A	N/A	.16	30 (48)		−0.378	−0.062	N/A	N/A		.71	
**Descriptive norms for substance use before sex**																
	Baseline to after the intervention	52 (85)	N/A	N/A	−0.274	−1.977 (51)	.053	N/A		N/A	N/A	N/A	N/A		N/A	
	Baseline to 1-mo follow-up	43 (70)	N/A	N/A	−0.541	−3.550 (42)	<.001	43 (69)		N/A	N/A	0.036	0.235 (42)		.82	
	Baseline to 3-mo follow-up	41 (67)	N/A	N/A	−0.410	−2.624 (40)	.01	42 (68)		N/A	N/A	0.079	0.514 (41)		.61	
	Baseline to 6-mo follow-up	39 (64)	N/A	N/A	−0.516	−2.951 (38)	.005	39 (63)		N/A	N/A	0.473	2.951 (38)		.003	

^a^n for each analysis was provided owing to missing data.

^b^CRAFFT: car, relax, alone, forget, friends, trouble.

^c^N/A: not applicable.

## Discussion

### Principal Findings

We found that it was feasible to recruit and retain adolescent participants for a randomized controlled trial comparing TWC with usual care in a primary care setting, despite the COVID-19 pandemic making recruitment and retention from primary care clinics uniquely challenging. All benchmarks for feasibility were met in this study, including recruitment and retention rates. TWC was completed in an average of 6.2 (SD 5.8) minutes, allowing for efficient integration into the clinic flow of the 2 pediatric primary care settings where adolescents engaged with the program in the waiting room or while waiting in an examination room to be seen by their provider. The majority of participants assigned to the intervention condition completed the full intervention (55/61, 90%), suggesting strong engagement with the content of the intervention. There was a fairly even split of participants who selected the various substance types or sexual risk scenarios, suggesting high interest in all of the content.

This study also examined preliminary secondary efficacy outcomes. The study was not adequately powered to assess differences; therefore, both statistical significance and effect sizes were examined. Given that small effect sizes would be expected for technology-based personalized normative feedback programs, small effect size differences were noted as potential items preliminary differences. We noted nonsignificant decreases in substance misuse risk among those in the TWC condition, resulting in a small effect size among those in the intervention condition, although these findings should be interpreted with caution. We found significant decreases in descriptive norms for substance use before sex among those in the TWC condition and significant increases among those in the control condition. Given that descriptive norms are associated with risk behaviors among youth and adults [10,12,13], changing descriptive norms associated with substance use before sex has the potential to affect youth behavior. Taken together, these findings suggest that a large randomized controlled trial to assess the efficacy of TWC is warranted.

Electronic interventions such as TWC can be easily disseminated with fidelity. They can be provided to patients using a clinic tablet or a patient’s own device. This may be particularly useful because technology-based substance use assessments take half the time of provider-led assessments [25], with equal sensitivity or specificity, within primary care settings among adolescents [26]. Adolescents also prefer self-report screening [16,27]. Adolescent preferences combined with provider time constraints to address all 12 required health promotion categories in the Bright Futures guidelines within a single visit [14] suggest a need for an intervention such as TWC. The web-based application format of TWC allows it to be accessed outside of the physical clinic, supporting implementation during telehealth visits. The ease of widespread dissemination within primary care clinics during routine preventive care appointments gives TWC the potential for significant public health impact.

### Strengths and Limitations

This study has several strengths, including the underrepresented population examined. Most of the participants identified as Black or African American (117/123, 95.1%) and were recruited from predominately Medicaid-serving pediatric clinics. Although this is a strength of this study, the results may not be generalizable to other racial and ethnic groups and clinic types. Another strength of this study was the rigorously designed pilot feasibility randomized controlled trial; however, as discussed previously, the primary outcome was feasibility, and therefore the study was not powered to detect differences in outcomes. Hence, the findings should be interpreted with caution and as preliminary, and future research should include an adequately powered study to conduct multivariate analyses. There was attrition over the 6-month study period. Therefore, although the feasibility benchmark for retention was met, retention was lower at the 6-month follow-up. Such attrition may be expected for a study that took place during the COVID-19 pandemic with little face-to-face interaction throughout the study. It is also important to note that challenges may arise when recruiting adolescents, especially those from underrepresented populations. The use of more culturally appropriate strategies as well as multiple outreach methods for follow-up may result in higher retention in future studies [28]. In addition, the number of potential participants approached was not recorded; therefore, we do not have this information to report for the study. We did not assess experiences of domestic violence in this study and, therefore, were unable to control for these histories in the analyses. Although our sample did include a small percentage of adolescents who identified as belonging to a gender minoritized group and a large percentage of participants who identified as belonging to a sexual minoritized group, more research is needed to ensure that this program and other programs targeting substance use, sexual assault, and sexual risk behaviors are acceptable and efficacious among adolescents belonging to sexual and gender minoritized groups. The assessment of health behaviors among adolescents in this study, such as substance use and condom use, relied on self-report. Therefore, it is possible that socially desirable responses were provided, particularly because the sample consisted of adolescents. Furthermore, this study was conducted during the COVID-19 pandemic, when changes in substance use among adolescents and likely sexual behavior were characteristically different than when social distancing measures were not in place. Therefore, future research should examine these findings under typical adolescent conditions. In TWC’s current form, it is not yet integrated with electronic medical records. Future research should consider integrating TWC with electronic medical records to communicate the results with providers.

### Conclusions

This study provides evidence for acceptable feasibility for recruitment and retention for a randomized controlled trial for TWC. The secondary outcomes revealed potential preliminary reductions in descriptive norms for substance use before sex and exposure to sexual assault as well as potential preliminary increases in bystander intervention intentions and condom use self-efficacy in the intervention condition. A large randomized controlled trial is needed to replicate and extend these findings and examine the mechanisms of change for substance use, sexual assault, and sexual risk behaviors among adolescents to ultimately improve public health in this area.

## Appendix

Multimedia Appendix 1 CONSORT Checklist.

## Abbreviations

CONSORT: Consolidated Standards of Reporting Trials
CRAFFT: car, relax, alone, forget, friends, trouble
SUD: substance use disorder
TWC: Teen Well Check

## References

[1] Miech RA, Johnston LD, Patrick ME, O'Malley PM, Bachman JG, Schulenburg JE (1975). Monitoring the future national survey results on drug use, 1975-2017: volume I, secondary school students. Institute for Social Research, The University of Michigan.

[2] Basile KC, Smith SG, Kresnow MJ, Khatiwada S, Leemis RW (2016). The national intimate partner and sexual violence survey: 2016/2017 report on sexual violence. Centers for Disease Control and Prevention.

[3] (2021). Youth risk behavior survey data summary and trends report: 2011-2021. Centers for Disease Control and Prevention.

[4] Kann L, McManus T, Harris WA, Shanklin SL, Flint KH, Queen B, Lowry R, Chyen D, Whittle L, Thornton J, Lim C, Bradford D, Yamakawa Y, Leon M, Brener N, Ethier KA (2018). Youth risk behavior surveillance - United States, 2017. MMWR Surveill Summ.

[5] Steele CM, Josephs RA (1990). Alcohol myopia: its prized and dangerous effects. Am Psychol.

[6] Ullman SE, Relyea M, Peter-Hagene L, Vasquez AL (2013). Trauma histories, substance use coping, PTSD, and problem substance use among sexual assault victims. Addict Behav.

[7] Saxton J, Rodda SN, Booth N, Merkouris SS, Dowling NA (2021). The efficacy of personalized normative feedback interventions across addictions: a systematic review and meta-analysis. PLoS One.

[8] Perkins HW, Berkowitz AD (1986). Perceiving the community norms of alcohol use among students: some research implications for campus alcohol education programming. Int J Addict.

[9] Borsari B, Carey KB (2003). Descriptive and injunctive norms in college drinking: a meta-analytic integration. J Stud Alcohol.

[10] Hoxmeier JC, Flay BR, Acock AC (2018). Control, norms, and attitudes: differences between students who do and do not intervene as bystanders to sexual assault. J Interpers Violence.

[11] Lewis MA, Litt DM, Cronce JM, Blayney JA, Gilmore AK (2014). Underestimating protection and overestimating risk: examining descriptive normative perceptions and their association with drinking and sexual behaviors. J Sex Res.

[12] Kantawong E, Kao TA, Robbins LB, Ling J, Anderson-Carpenter KD (2022). Adolescents' perceived drinking norms toward alcohol misuse: an integrative review. West J Nurs Res.

[13] Pedersen ER, Osilla KC, Miles JN, Tucker JS, Ewing BA, Shih RA, D'Amico EJ (2017). The role of perceived injunctive alcohol norms in adolescent drinking behavior. Addict Behav.

[14] Hagan JF Jr, Shaw JS, Duncan PM (2017). Bright Futures: Guidelines for Health Supervision of Infants, Children, and Adolescents. 4th edition.

[15] Champion KE, Parmenter B, McGowan C, Spring B, Wafford QE, Gardner LA, Thornton L, McBride N, Barrett EL, Teesson M, Newton NC, Health4Life team (2019). Effectiveness of school-based eHealth interventions to prevent multiple lifestyle risk behaviours among adolescents: a systematic review and meta-analysis. Lancet Digit Health.

[16] Gibson EB, Knight JR, Levinson JA, Sherritt L, Harris SK (2021). Pediatric primary care provider perspectives on a computer-facilitated screening and brief intervention system for adolescent substance use. J Adolesc Health.

[17] Gilmore AK, Mosley EA, Oesterle DW, Ridings LE, Umo I, Hutchins A, Gooding HC, Wallis E, Levy S, Ruggiero K, Kaysen D, Danielson CK, Self-Brown S (2023). Teen Well Check: an e-health prevention program for substance use, sexual assault, and sexual risk behaviors for adolescents in primary care. Eur J Psychotraumatol.

[18] Eldridge SM, Chan CL, Campbell MJ, Bond CM, Hopewell S, Thabane L, Lancaster GA, PAFS consensus group (2016). CONSORT 2010 statement: extension to randomised pilot and feasibility trials. BMJ.

[19] Knight JR, Shrier LA, Bravender TD, Farrell M, Vander Bilt JV, Shaffer HJ (1999). A new brief screen for adolescent substance abuse. Arch Pediatr Adolesc Med.

[20] Teen brain development. National Institute on Drug Abuse.

[21] Levy S, Weiss R, Sherritt L, Ziemnik R, Spalding A, Van Hook S, Shrier LA (2014). An electronic screen for triaging adolescent substance use by risk levels. JAMA Pediatr.

[22] Shenoi RP, Linakis JG, Bromberg JR, Casper TC, Richards R, Mello MJ, Chun TH, Spirito A, Pediatric Emergency Care Applied Research Network (2019). Predictive validity of the CRAFFT for substance use disorder. Pediatrics.

[23] Bedard-Gilligan MA, Dworkin ER, Kaysen D, Ojalehto HJ, Stappenbeck CA, Lindgren KP (2022). A pilot study on the feasibility, acceptability, and preliminary efficacy of a brief text message intervention for co-occurring alcohol misuse and PTSD symptoms in a community sample. J Anxiety Disord.

[24] Bricca A, Swithenbank Z, Scott N, Treweek S, Johnston M, Black N, Hartmann-Boyce J, West R, Michie S, de Bruin M (2022). Predictors of recruitment and retention in randomized controlled trials of behavioural smoking cessation interventions: a systematic review and meta-regression analysis. Addiction.

[25] Harris SH, Louis-Jacques J, Knight JR, Brown RT, Ryan S (2014). Screening and brief intervention for alcohol and other abuse. AM:STARs: Substance Use and Abuse Among Adolescents, Vol. 25, No. 1: Adolescent Medicine: State of the Art Reviews AM:STARs: Substance Use and Abuse Among Adolescents, Vol. 25, No. 1: Adolescent Medicine: State of the Art Reviews.

[26] Harris SK, Knight JR Jr, Van Hook S, Sherritt L, Brooks TL, Kulig JW, Nordt CA, Saitz R (2016). Adolescent substance use screening in primary care: validity of computer self-administered versus clinician-administered screening. Subst Abus.

[27] Knight JR, Harris SK, Sherritt L, Van Hook S, Lawrence N, Brooks T, Carey P, Kossack R, Kulig J (2007). Adolescents' preference for substance abuse screening in primary care practice. Subst Abus.

[28] Dickerson DL, Parker J, Johnson CL, Brown RA, D'Amico EJ (2021). Recruitment and retention in randomized controlled trials with urban American Indian/Alaska native adolescents: challenges and lessons learned. Clin Trials.

